# Effects of repetitive peripheral magnetic stimulation on poststroke ankle spasticity: a double-blind randomized controlled trial

**DOI:** 10.55730/1300-0144.6197

**Published:** 2026-02-01

**Authors:** Hüseyin Oğuzhan ASLANTAŞ, Şehim KUTLAY, Haydar GÖK, Semra ÖZKAN, Seçilay GÜNEŞ, Atilla Halil ELHAN

**Affiliations:** 1 Department of Physical Medicine and Rehabilitation, Faculty of Medicine, Ankara University, Ankara, Turkiye; 2 Department of Biostatistics, Faculty of Medicine, Ankara University, Ankara, Turkiye

**Keywords:** Stroke, spasticity, repetitive peripheral magnetic stimulation, H-reflex (Hoffmann), electromyography

## Abstract

**Background/aim:**

Repetitive peripheral magnetic stimulation (rPMS) is a noninvasive, painless therapy aimed at improving spasticity, with a proposed mechanism that involves neuromodulation through somatosensory and proprioceptive afferent stimulation. The present study evaluates the effectiveness of rPMS on ankle plantar flexor spasticity in participants with stroke.

**Materials and methods:**

Included in this prospective, double-blind, randomized, sham-controlled study were 50 participants with poststroke ankle plantar flexor spasticity graded 1–3 according to Modified Ashworth Scale (MAS). Participants were randomized into rPMS (n = 25) and sham (n = 25) groups. All participated in a conventional rehabilitation program, while the rPMS group received 10 rPMS sessions over 2 weeks and the sham group received sham rPMS. Assessments at baseline, posttreatment (2nd week), and follow-up (4th week) included MAS, Modified Tardieu Scale (MTS), Barthel Index (stair/mobility), and electrophysiological H/M ratio.

**Results:**

A total of 46 participants completed the study. No adverse events related to the stimulation were reported. The rPMS group showed significant improvements in MAS score, MTS angle and grade, and Barthel Index, while no significant changes were observed in the sham group. A significantly greater proportion of participants in the rPMS group (40%) demonstrated MAS score reduction compared to the sham group (4.8%) (p = 0.004). Between-group analysis favored rPMS for MAS and MTS posttreatment and at follow-up. No significant changes in H/M ratios were observed within or between the groups.

**Conclusion:**

rPMS can be a valuable adjunct to a traditional rehabilitation program, potentially improving poststroke spasticity. The reductions recorded in MAS and MTS scores in the absence of electrophysiological changes suggest that rPMS may reduce the biomechanical rather than neural component of spasticity. There is a need to define optimal treatment protocols.

## 1. Introduction

Poststroke spasticity is a significant clinical problem with substantial consequences for patients, caregivers, and healthcare systems, and potential complications for functional rehabilitation. The prevalence of poststroke spasticity ranges from 17% to 43% in stroke survivors more than 3 months poststroke [1]. Spasticity management requires a comprehensive, patient-specific, and team-based neurorehabilitation program including nonpharmacological, pharmacological, and surgical interventions. Nonpharmacological interventions include such broad-ranging modalities as electrical stimulation, extracorporeal shockwave therapy, posture or physical management, transfer, dynamic movement, stretching, splinting, and casting. These interventions can be combined within a multimodal therapy program as a first-line treatment [2].

Repetitive peripheral magnetic stimulation (rPMS) is a noninvasive, painless physical therapy modality with relatively few side effects. In rPMS, a high-intensity electromagnetic field is created that can stimulate peripheral nerves and muscles. It has been hypothesized to modulate neural excitability through somatosensory and proprioceptive pathways [3].

A review of the literature reveals that studies investigating the effects of rPMS on spasticity have focused mainly on the upper extremities [3–12]. To the best of our knowledge, there has been only one randomized controlled trial to date investigating the efficacy of rPMS on lower limb spasticity in patients with stroke, and the sample size was quite small [13]. The present study aims to address this significant gap in knowledge.

The present study evaluates the effectiveness of rPMS for the treatment of ankle plantar flexor muscle spasticity in participants with stroke, and investigates its potential underlying mechanisms through electrophysiological assessment. It is hypothesized that 10 sessions of rPMS applied to the ankle plantar flexor muscles in participants with poststroke spasticity, as an adjunct to conventional rehabilitation, will reduce spasticity, as measured by the Modified Ashworth Scale (MAS).

## 2. Materials and methods

The study was designed as a prospective, randomized, double-blind (investigator, participant), sham-controlled clinical trial. A total of 189 patients with stroke-related ankle plantar flexor spasticity were screened at the Neurorehabilitation Unit of the Department of Physical Medicine and Rehabilitation, Faculty of Medicine, Ankara University. After the application of the inclusion criteria, written informed consent was provided by those who agreed to take part in the study.

The inclusion criteria were: age ≥18 years; ankle plantar flexor spasticity grade 1–3 according to the MAS; medically stable condition (no uncontrolled systemic disease such as hypertension, diabetes, cardiac disease, etc.); and no history of previous rPMS treatment. Excluded from the study were those with ankle contracture; malignancy; vascular issues, such as deep venous thrombosis; peripheral artery disease; local inflammation/infection at the treatment site; cardiac pacemakers; drug pumps; cochlear implants; metal implants in the treatment area; nonunion fractures in the treatment area; bleeding disorders; history of botulinum toxin; phenol or alcohol injections within the last 6 months; previous surgical treatments of calf muscles; and changes to doses of oral anti-spastic drugs within the last 6 months.

Ethical approval was obtained from the Clinical Research Ethics Committee of Ankara University Faculty of Medicine, as well as the Turkish Medicine and Medical Device Agency. This study was registered on the Australian and New Zealand Clinical Trials website (trial ID: ACTRN12622000266763, date registered: 14/02/2022), and was conducted in accordance with the Declaration of Helsinki and the International Conference on Harmonization Good Clinical Practice Guidelines.

After screening and evaluation, the eligible participants were assigned to either the rPMS or sham group. All participants attended a rehabilitation program that included stretching, strengthening, range of motion, and neurophysiological exercises, and occupational therapy (five sessions per week, 45–60 min a day, total 2 weeks) in the Neurorehabilitation Unit.

**Figure 1:**
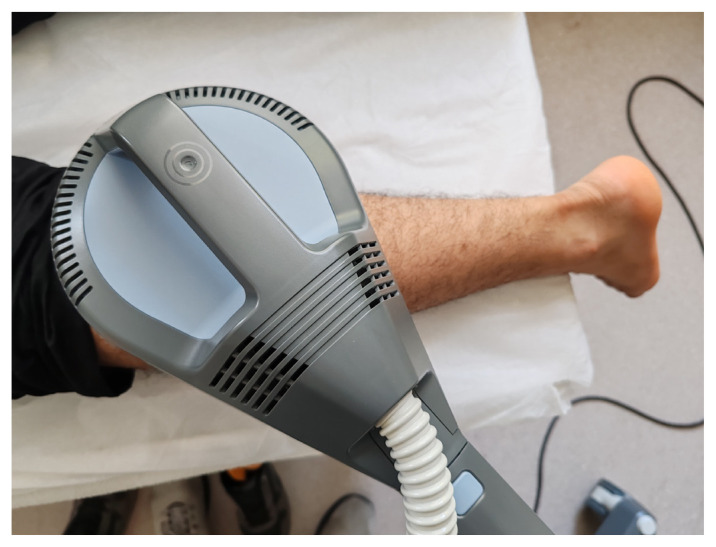
Participant positioning and probe placement during rPMS treatment.

rPMS and sham rPMS were administered to the ankle plantar flexor muscles on the hemiplegic side in five sessions/week for a period of 2 weeks (total 10 sessions). The patient was positioned in the prone position, and the probe was placed over the ankle plantar flexor muscle (Figure 1). The rPMS was applied using a BTL-6000TM Super Inductive System Elite device (United Kingdom), with “Spasticity reduction protocol” selected from the device menu. The protocol was applied in six different phases in which different waveforms and frequency modulations were applied. The entire protocol lasted 10 min. The frequencies applied were in the range of 25–150 Hertz, the pulse duration was 280 μs, and the wave was sinusoidal biphasic. Stimulation intensity was increased until visible contraction was observed at the ankle plantar flexor muscle. A similar device and patient positioning were used in the sham group. The device was switched on but no stimulation was applied, and the participants were exposed to the recorded sound of a typical rPMS treatment session for 10 min. All participants were observed closely by a physiotherapist throughout the treatment.

All participants were assessed by the same physician, who was blinded to the randomization and treatment procedures. The patients were evaluated at baseline, posttreatment (2nd week), and at follow-up (4th week). The primary outcome measure was MAS [14]. For convenience, MAS grade 1+ was graded as point 2, and grades 2, 3, and 4 as 3, 4, and 5, respectively, during the statistical analysis. Secondary outcomes were the Modified Tardieu Scale (MTS) spasticity angle and grade, the Modified Barthel Index (BI) lower extremity score (sum of stair and mobility scores), and the Hmax/Mmax ratio [15]. Greater spasticity is associated with higher MAS and MTS spasticity grades and a lower MTS spasticity angle. Maximal H-reflex and M-wave responses were collected at increasing stimulation intensities using a “Keypoint-Dantec” (Denmark) electromyography device. A decrease in the Hmax/Mmax ratio indicates a reduction in spasticity [15].

After screening and evaluation, all participants were randomly assigned to the rPMS or sham groups using a block randomization method to mitigate the effect of any potential differences in the sample and to reduce the risk of unequal group sizes in the event of the planned sample size not being attained. Random Allocation Software (Ver. 1.0.0) was used to allocate the participants into groups with the block size set to 2. Allocation concealment was maintained by using sequentially numbered envelopes prepared according to a computer-generated random numbers table. The physicians performing the clinical evaluations and conducting the electrophysiological examinations, as well as all participants in the sham and rPMS groups, were blinded to the treatment allocation.

The primary outcome was the Modified Ashworth Scale (MAS) score. To ensure a targeted power of at least 80% with a significance level of 0.05 using a two-sided Mann–Whitney U test, a sample size of 21 participants per group (total n = 42) is required, yielding an actual power of 81%. This calculation was based on detecting a difference of 0.36 between the groups, assuming an expected mean of 0.40 for the intervention group, 0.04 for the sham group, and a standard deviation of 0.5 for both groups. The required sample size was thus calculated as 42, and considering a drop-out rate of 20%, the final sample size was set at 50, with 25 participants assigned to each group.

Descriptive statistics were presented as frequencies and percentages for categorical variables. Normally distributed continuous variables were presented as mean ± standard deviation, while nonnormally distributed variables were presented as median (minimum–maximum, interquartile range: 25th–75th percentiles). Normality was assessed using a Shapiro–Wilk test. Since parametric assumptions were not fully met, nonparametric statistical methods were applied. Between-group comparisons of categorical variables were performed using the chi-square or Fisher’s exact test as appropriate. Differences in nonnormally distributed continuous variables and ordinal variables between two independent groups were evaluated using the Mann–Whitney U test. For comparisons across three repeated measurements, Friedman’s two-way analysis of variance by ranks was applied. When the p-value from the Friedman test statistics was statistically significant, Dunn’s test was used to understand which measurement band differed from which others. The Bonferroni correction was applied to control the Type I error rate for within- and between-group comparisons. A p-value of <0.05 was considered statistically significant. All statistical analyses were conducted using IBM SPSS Statistics, version 30.0.

## 3. Results

**Figure 2:**
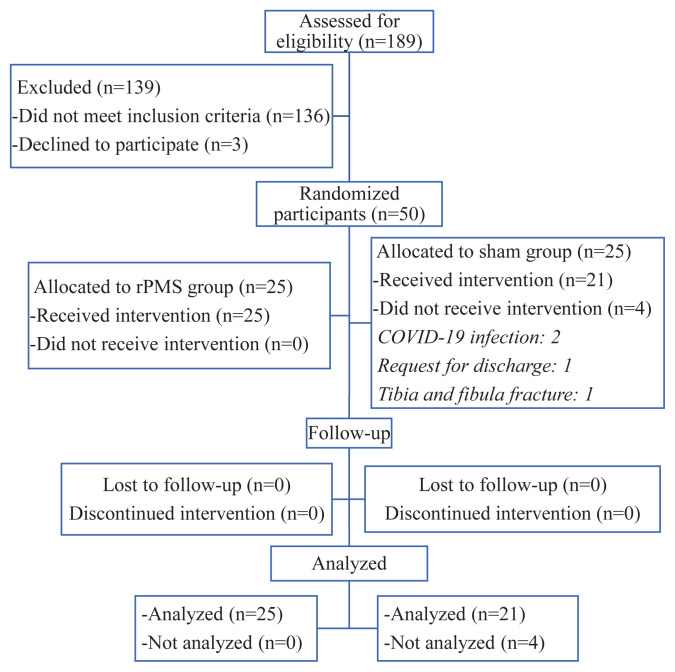
CONSORT flow diagram.

Of the 189 patients assessed for eligibility, 136 did not meet the inclusion criteria and three declined to take part. The study thus commenced with 50 participants, who were randomly and equally assigned to the rPMS and sham groups. Four members of the sham group subsequently dropped out of the study: one after sustaining a fracture of the tibia and fibula in a traffic accident; one who requested a voluntary discharge; and two who contracted COVID-19 at the beginning of treatment. Consequently, the study was completed with a total of 46 patients (Figure 2). The sociodemographic and clinical data of the study population are presented in Table 1. The mean age was 64 ± 16 (22–87) years in the rPMS group and 59 ± 12 (33–74) years in the sham group. The mean time since the stroke was longer in the sham group than in the rPMS group (55.14 ± 81.25 vs 27.12 ± 65.06 months, respectively).

A comparison of changes in clinical parameters within groups over time is presented in Tables 2 and 3. No significant improvements were observed in the MAS scores (pretreatment [T1] and follow-up [T3] mean ± SD values: T1: 2.30 ± 0.76; T3: 1.87 ± 0.69; p = 0.001), MTS spasticity angle (R2-R1) (T1: 13.09 ± 8.41; T3: 10.57 ± 6.99; p = 0.017), MTS spasticity grade (T1: 2.74 ± 0.69; T3: 2.48 ± 0.73; p = 0.002), or BI (sum of the stair and mobility sections) (T1: 11.09 ± 10.76; T3: 13.48 ± 10.49; p = 0.004) of the rPMS group. The changes in the sham group did not reach statistical significance in any of these parameters. For the rPMS group, post hoc analysis revealed that the decrease in the MAS score occurred between the pretreatment and follow-up MAS scores (p = 0.033). Subsequently, the direction of change in the posttreatment MAS scores of the study groups relative to their pretreatment score was calculated. A significantly greater percentage of subjects in the rPMS group (40%) than the sham group (4.8%) observed a reduction in their MAS scores (p = 0.004). The improvement in the angle of muscle reaction (MTS R1) occurred posttreatment and at follow-up (p = 0.033 and p = 0.001, respectively). Other clinical measures did not show a significant difference on post hoc analysis (Tables 2 and 3).

Between-group analysis revealed that the improvements in the MAS scale and MTS spasticity grade were greater in the rPMS group after treatment and during follow-up (p = 0.008, p = 0.035, p = 0.010, and p = 0.006, respectively) (Tables 4 and 5). There was no significant change in the H/M ratio of the affected side within the rPMS and sham groups (p = 0.332 and p = 0.692, respectively), and the two groups were comparable in terms of changes in H/M ratios (p >0.05). Notably, no adverse events were reported related to the stimulation and all participants tolerated the rPMS treatment well.

## 4. Discussion

This randomized controlled study demonstrated that 10 sessions of rPMS when applied in combination with a conventional rehabilitation program were effective in reducing ankle plantar flexor spasticity in participants with stroke, with no reported side effects.

A review of the existing literature reveals that studies of the effects of rPMS on spasticity have focused more on the upper extremities than the lower extremities [3–12]. Furthermore, only four of the studies evaluating the effects of rPMS on the lower extremities were conducted in participants with stroke [13,16–18]. The remaining studies included participants with various neurological conditions, including multiple sclerosis (MS), spinal cord injury, and cerebral palsy [19–25]. Only one of these studies was a randomized controlled trial [13], and it was limited by its small sample size (18 participants with stroke: 9 in the rPMS group, 9 in the sham group) and the administration of rPMS to the tibialis anterior muscle in only a single session. Unlike the present study, the authors sought to reduce spasticity indirectly through the strengthening of the antagonist muscle, and they did not measure spasticity using the widely used assessment as the MAS or MTS, making direct comparison with the present study difficult.

The population included in the present study comprised individuals with both subacute and chronic stroke, resulting in heterogeneity in time since stroke onset values and, consequently, in spasticity duration. This variability reflects a common challenge in rehabilitation research. Spasticity may arise predominantly from neural hyperreflexia in the subacute phase and from biomechanical muscle remodeling in the chronic phase; however, its management remains clinically relevant across the entire stroke continuum. Given that spasticity constitutes a substantial clinical burden in both subacute and chronic stages, our evaluation of a broad patient spectrum allowed us to evaluate the practical utility of rPMS in addressing this persistent condition across different phases of recovery.

In the present study, rPMS treatment administered together with a conventional rehabilitation program was observed to have a spasticity-reducing effect, which was reflected in significant decreases in the MAS score, MTS spasticity grade and spasticity angle. This result is consistent with the findings of previous studies involving stroke patients [5,7,9,10,12,17,18]. It should be noted that these earlier studies tended to have small sample sizes and did not include a power analysis, and some lacked a control group for comparison. In a number of the studies that used MTS as an assessment tool, only a single component was measured—either spasticity angle or spasticity grade [6,7,24], while in the present study, both components were evaluated together.

The results of the current study also align with those of various studies focused on other neurological conditions in which rPMS treatment has been reported to improve upper [8,19] and lower extremity spasticity [19,20,22,23] with a reduction in the MAS scores. In contrast, our findings differ from two studies in which no significant changes in MAS scores were reported in participants with stroke, investigating upper extremity and lower extremity spasticity, respectively [11,16]. In the upper limb trial, in which there was no control group, rPMS was applied to six distinct spastic muscles in 13 participants with stroke in a single session lasting 2 min per muscle. This single-session procedure and brief application duration may have made it difficult to detect an effect [11].

A further study assessing the impact of rPMS on gait in seven individuals with stroke revealed spasticity in various lower extremity muscle groups in five of the participants; however, there was no significant change in MAS scores after rPMS treatment [16]. The absence of significant effects on spasticity in this study cannot be generalized.

In the present study, the sum of the BI stair and mobility section scores was used to evaluate the effects of rPMS treatment on lower extremity functional status. To mitigate the potential ceiling effect of the full BI and to enhance its sensitivity to lower-limb interventions, our analysis focused specifically on the subitems directly related to lower extremity function and mobility (e.g., transfers and ambulation). By isolating these components, we aimed to exclude “noise” from unrelated activities—such as feeding—thereby providing a more targeted assessment of how reductions in ankle spasticity translate into functional gait and postural transitions. While a significant improvement was observed within the rPMS group, no superiority of rPMS over the sham group was demonstrated. Our review of the literature revealed several studies in which the BI was used with sole focus on the upper extremities, while no application on the lower extremities has been reported to date, preventing the comparison of our results with existing literature [9,10]. It is important to note that all participants continued their conventional physiotherapy programs concurrently with the spasticity intervention, and this background therapy likely contributed to the improvements in the activities of daily living observed in both groups. Our findings suggest that rPMS is effective as an adjunctive therapy within a comprehensive rehabilitation framework.

In the present study, H/M ratios were measured on both the affected and unaffected sides. However, the reduction in spasticity observed after rPMS treatment was not reflected in changes in the H/M ratio. A review of the literature revealed no studies evaluating the effect of rPMS on participants with stroke using the H/M ratio, and studies evaluating the H/M ratio in other neurological conditions and healthy participants have produced contradictory results. Two studies of patients with MS in which the coil was placed para-vertebrally and 1–7 sessions of rPMS were applied reported findings different to ours. Specifically, a reduction in H reflex amplitude and H/M ratio was observed in their rPMS groups, but no significant difference compared to their control groups [21,22]. In one of these studies it was stated that the changes in the H reflex amplitude may have been influenced by factors such as coil placement, magnetic field intensity, and the frequency of the current applied [21]. The differences in the results of these two studies with the present study may be due to the use of different coil placements and treatment protocols. In studies conducted on healthy participants, rPMS was applied to the peroneal nerve, radial nerve, or calf muscles of the participants in a single session, leading to a decrease in the compound muscle action potential triggered by the Achilles reflex, while the amplitude of the H reflex decreased or remained unchanged and the H/M ratio did not change. The results of these studies regarding the effect of rPMS treatment on spinal excitability are conflicting [26–30]. In the present study, the reduction in spasticity observed in evaluations using the MAS and MTS, together with the absence of electrophysiological differences, suggest that rPMS treatment may reduce the biomechanical component of spasticity through its local effects on the muscles rather than spinal excitability. The dissociation between reflex excitability and clinical resistance observed in the present study is consistent with the “spasticity paradox” described by Burne et al. [31], whereby mechanical stiffness of the muscle-tendon unit often predominates over neural hyperreflexia. As highlighted by Dietz and Sinkjaer [32], clinical improvements frequently arise from alterations in nonneural components, which the H-reflex is inherently unable to capture because it bypasses the gamma loop and muscle spindle sensitivity. There are also earlier studies suggesting that the H/M ratio does not correlate with clinical spasticity scales [33–36].

Significant variations in rPMS treatment parameters in different studies have been identified, including the application site (on the muscle, spinal-paraspinal region, or peripheral nerve), frequency, intensity, duration, coil type, number of pulses, pulse duration, number of sessions, and duty cycle. It can thus be understood that there is as yet no standardized rPMS protocol for the treatment of spasticity. Previous studies focused on various neurological diseases have applied protocols ranging from 1 to 20 sessions, using round or figure-eight coils, biphasic current, pulse durations of 240–400 μs, frequencies of 1–150 Hz, intensities up to 42–45% of the device’s maximum output or 0–120% above the motor threshold level, and with 600–10,000 pulses per session for durations of 3–40 min [3–13,16,19–23]. In the present study, 10 × 10 min rPMS sessions were applied in six sections to the ankle plantar flexor muscles using a round coil. A sinusoidal biphasic current type was used with a 280-μs pulse duration and a frequency range of 25–150 Hz, and with stimulus intensity set just above the threshold to induce visible muscle contraction. This difference in treatment protocols makes comparison challenging.

The present study has several strengths and some limitations. The major strengths of this study include its double-blind, randomized design, its active sham stimulation, its a priori sample size determination, and its comprehensive evaluation of spasticity using the MAS, MTS spasticity angle, and MTS spasticity grade together. To the best of our knowledge, this is the first randomized, double-blind, controlled study to date to evaluate the effectiveness of rPMS on lower extremity spasticity in participants with stroke using an electrophysiological method as an outcome parameter.

One limitation of the study is its inclusion of participants with both subacute and chronic stroke, which resulted in variability in the time since stroke onset value and, consequently, in spasticity duration. The varied stroke durations among the participants and the small sample size, which precluded a subgroup analysis, may have influenced the study’s findings. Another limitation was the use of a single treatment protocol during the rPMS application, which was the spasticity treatment scheme recommended by the device manufacturer. Therefore, the effects of various parameters, including coil type, placement location, application duration, frequency, and intensity, on rPMS treatment remain unanswered. Although the stimulation parameters used in this study are consistent with those commonly reported in the literature, the specific pulse waveforms and coil geometries of the device may limit the direct generalizability of our findings to all rPMS systems, and the results should thus be interpreted with this in mind. Future studies should prioritize multicenter trials and protocol optimization, which may advise the establishment of standardized, device-independent clinical guidelines for spasticity management. As a final limitation, the long-term effects of the protocol cannot be evaluated as the final examination was carried out 2 weeks after the end of therapy.

The findings of the present study highlight the promising role of rPMS as an adjunct to conventional rehabilitation methods in reducing ankle plantar flexor spasticity among participants with stroke. With no reported side effects, rPMS can be considered a safe and effective physical therapy modality. However, to fully realize its benefits, further studies are needed to elucidate its long-term effects and to determine the optimal stimulation parameters for different patient profiles.

## Figures and Tables

**Table 1:** Sociodemographic and clinical characteristics of participants.

	rPMS group (n=25)	Sham group (n=21)

**Age (years)**		
Mean ± SD	64 ± 16	59 ± 12
(min–max)	(22–87)	(33–74)

**Sex, n (%)**		
Male	11 (44.0%)	12 (57.1%)
Female	14 (56.0%)	9 (42.9%)

**Time since stroke (months)**		
Mean ± SD	27.52 ± 66.01	55.94 ± 82.44
median (IQR)	3.93 (27–48)	25.33 (64–421)
(min–max)	(0.16–316.12)	(0.26–291.75)

**Stroke etiology, n (%)**		
Ischemic	16 (64.0%)	13 (61.9%)
Hemorrhagic	7 (28.0%)	6 (28.6%)
Ischemic + Hemorrhagic	2 (8.0%)	2 (9.5%)

**Dominant hand, n (%)**		
Right	22 (88.0%)	19 (90.5%)
Left	3 (12.0%)	2 (9.5%)

rPMS: Repetitive peripheral magnetic stimulation, n: number, SD: Standard deviation, IQR: interquartile range.

**Table 2:** Comparison of changes in clinical parameters within groups *(MAS and MTS angles)*.

		rPMS group (n = 25)						Sham group (n = 21)				
		Mean±SD	Median	Min–max	P25–P75	P^a^	P^b^	Mean±SD	Median	Min–max	P25–P75	P^a^
**MAS**	T1	2.30 ± 0.76	2.00	1–4	2–3	**0.001**	0.102 **0.033** 1.000	2.39 ± 0.98	2.00	1–4	2–3	0.247
	T2	1.96 ± 0.77	2.00	1–4	1–2			2.39 ± 1.04	2.00	1–4	2–3	
	T3	1.87 ± 0.69	2.00	1–3	1–2			2.28 ± 0.96	2.00	1–4	2–3	
**MTS R1**	T1	72.09 ± 11.72	70.00	50–89	64–82	**0.001**	**0.033** **0.001** 0.867	75.00 ± 9.18	76.00	56–92	69–80	0.368
	T2	75.57 ± 10.64	79.00	50–90	70–85			75.44 ± 7.05	75.00	62–90	70–80	
	T3	76.22 ± 10.85	81.00	51–91	70–85			74.78 ± 6.96	73.00	62–90	70–78	
**MTS R2**	T1	85.17 ± 12.52	90.00	58–108	80–91	**0.045**	0.472 0.143 1.000	85.33 ± 9.49	85.00	69–100	80–92	0.695
	T2	86.30 ± 12.84	90.00	55–109	80–92			84.78 ± 8.00	83.50	70–100	80–91	
	T3	86.78 ± 12.28	90.00	60–109	81–94			84.83 ± 7.79	83.00	72–100	80–91	
**R2-R1**	T1	13.09 ± 8.41	10.00	3–35	8–15	**0.017**	0.059 0.102 1.000	10.33 ± 6.51	8.00	2–25	7–18	0.747
	T2	10.74 ± 6.61	10.00	1–28	5–12			9.33 ± 6.43	8.00	3–28	5–15	
	T3	10.57 ± 6.99	9.00	2–28	5–11			10.06 ± 6.44	8.50	2–25	6–18	

rPMS: Repetitive peripheral magnetic stimulation; MAS: Modified Ashworth Scale; MTS: Modified Tardieu Scale; R2-R1: Modified Tardieu Scale spasticity angle; SD: Standard deviation; P25–P75: 25th–75th percentile; T1: Pretreatment; T2: Posttreatment; T3: Follow-up; P^a^: Within-group change; P^b^: Post hoc comparison of T1–T2, T1–T3 and T2–T3 respectively. Significance values have been adjusted by the Bonferroni correction for multiple tests.

**Table 3:** Comparison of changes in te clinical parameters *(MTSg, Barthel, H/Ma)* within the respective groups *(MTS spasticity grade, Barthel Index and H/M ratio)*.

		rPMS group (n = 25)						Sham group (n = 21)				
		Mean±SD	Median	Min–max	P25–P75	P^a^	P^b^	Mean±SD	Median	Min–max	P25–P75	P^a^
**MTSg**	T1	2.74 ± 0.69	3.00	2–4	2–3	**0.002**	0.609 0.269 1.000	2.72 ± 0.89	3.00	1–4	2–4	0.097
	T2	2.57 ± 0.66	2.00	2–4	2–3			2.83 ± 0.92	3.00	1–4	2–4	
	T3	2.48 ± 0.73	2.00	1–4	2–3			2.78 ± 0.88	3.00	1–4	2–4	
**Barthel**	T1	11.09 ± 10.76	10.00	0–25	0–23	**0.004**	0.867 0.413 1.000	18.33 ± 7.67	20.00	0–25	15–25	0.135
	T2	13.26 ± 10.40	15.00	0–25	0–25			18.89 ± 7.58	22.50	0–25	15–25	
	T3	13.48 ± 10.49	15.00	0–25	0–25			18.89 ± 7.58	22.50	0–25	15–25	
**H/Ma**	T1	0.35 ± 0.35	0.14	0.02–1.32	0.07–0.56	0.332		0.54 ± 0.82	0.31	0.03–3.70	0.24–0.53	0.692
	T2	0.27 ± 0.23	0.18	0.02–0.85	0.09–0.42		0.36 ± 0.26	0.32	0.04–0.95	0.21–0.50		
	T3	0.25 ± 0.23	0.17	0.01–0.79	0.09–0.45		0.34 ± 0.27	0.27	0.04–0.92	0.10–0.48		

rPMS: Repetitive peripheral magnetic stimulation; MTSg: Modified Tardieu Scale spasticity grade; Barthel: Sum of Barthel Index stair and mobility sections; H/Ma: Hmax/Mmax ratio measured from the affected side; SD: Standard deviation; P25–P75: 25th–75th percentile; T1: Pretreatment; T2: Posttreatment; T3: Follow-up; P^a^: Within-group change; P^b^: Post hoc comparison of T1-T2, T1-T3 and T2-T3 respectively. Significance values have been adjusted by the Bonferroni correction for multiple tests.

**Table 4:** Comparison of changes in the clinical parameters between the groups *(MAS and MTS angles)*.

		rPMS group (n = 25)				Sham group (n = 21)				
		Mean±SD	Median	Min–max	P25–P75	Mean±SD	Median	Min–max	P25–P75	p
**MAS**	T1-T2	−0.4 ± 0.50	0.00	−1 to 0	−1 to 0	0.05 ± 0.38	0.00	−1 to 1	0–0	**0.008**
	T1-T3	−0.48 ± 0.51	0.00	−1 to 0	−1 to 0	−0.10 ± 0.44	0.00	−1 to 1	0–0	**0.035**
	T2-T3	−0.08 ± 0.28	0.00	−1 to 0	0–0	−0.14 ± 0.36	0.00	−1 to 0	0–0	1.0
**MTS R1**	T1-T2	3.68 ± 6.74	5.00	−9 to 25	1–7	0.48 ± 5.16	2.00	−12 to 7	−3 to 5	0.267
	T1-T3	4.32 ± 6.65	4.00	−8 to 26	2–7	−0.19 ± 5.79	2.00	−15 to 7	−3 to 4	0.087
	T2-T3	0.64 ± 3.24	0.00	−10 to 10	0–1	−0.67 ± 2.35	0.00	−6 to 3	−2 to 0	0.319
**MTS R2**	T1-T2	1.12 ± 4.93	0.00	−15 to 12	0–2	−0.38 ± 4.44	0.00	−15 to 5	−1 to 2	0.814
	T1-T3	1.56 ± 4.87	1.00	−10 to 12	0–4	−0.33 ± 4.80	0.00	−15 to 5	0–2	1.0
	T2-T3	0.44 ± 2.92	0.00	−5 to 10	0–1	0.05 ± 2.33	0.00	−7 to 7	0–0	1.0
**R2-R1**	T1-T2	−2.56 ± 5.18	−2.00	−14 to 10	−5 to 0	−0.86 ± 3.99	0.00	−10 to 7	−4 to 2	0.516
	T1-T3	−2.72 ± 4.15	−2.00	−14 to 5	−5 to 0	−0.14 ± 3.44	0.00	−5 to 7	−2 to 2	0.152
	T2-T3	−0.16 ± 2.62	0.00	−7 to 5	0–1	0.71 ± 3.48	0.00	−4 to 12	−1 to 1	1.0

rPMS: Repetitive peripheral magnetic stimulation; MAS: Modified Ashworth Scale; MTS: Modified Tardieu Scale; R2-R1: Modified Tardieu Scale spasticity angle; SD: Standard deviation; P25–P75: 25th–75th percentile; T1: Pretreatment; T2: Posttreatment; T3: Follow-up. Significance values have been adjusted by the Bonferroni correction for multiple tests. Bold values indicate statistical significance.

**Table 5:** Comparison of changes in clinical parameters between groups *(MTS spasticity grade, Barthel Index and H/M ratio)*.

		rPMS group (n = 25)				Sham group (n = 21)				
		Mean±SD	Median	Min–max	P25-P75	Mean±SD	Median	Min–max	P25-P75	p
**MTSg**	T1-T2	−0.24 ± 0.44	0.00	−1 to 0	0–0	0.14 ± 0.36	0.00	0–1	0–0	**0.010**
	T1-T3	−0.32 ± 0.48	0.00	−1 to 0	−1 to 0	0.10 ± 0.30	0.00	0–1	0–0	**0.006**
	T2-T3	−0.08 ± 0.28	0.00	−1 to 0	0–0	−0.05 ± 0.22	0.00	−1 to 0	0–0	1.0
**Barthel**	T1-T2	2.00 ± 4.33	0.00	0–15	0–0	0.50 ± 1.54	0.00	0–5	0–0	0.880
	T1-T3	2.80 ± 5.02	0.00	0–15	0–5	0.50 ± 1.54	0.00	0–5	0–0	0.305
	T2-T3	0.8 ± 3.12	0.00	0–15	0–0	0.00 ± 0.00	0.00	0–0	0–0	0.602
**H/Ma**	T1-T2	−0.07 ± 0.27	−0.01	−0.91 to 0.29	−0.095 to 0.055	−0.19 ± 0.77	−0.02	−3.42 to 0.18	−0.106 to 0.065	1.0
	T1-T3	−0.1 ± 0.22	−0.01	−0.66 to 0.11	−0.16 to 0.04	−0.19 ± 0.86	0.02	−3.634 to 0.39	−0.07 to 0.12	0.675
	T2-T3	−0.01 ± 0.15	0.00	−0.5 to 0.27	−0.06 to 0.03	−0.02 ± 0.13	0.01	−0.43 to 0.21	−0.03 to 0.04	1.0

rPMS: Repetitive peripheral magnetic stimulation; MTSg: Modified Tardieu Scale spasticity grade; Barthel: Sum of Barthel Index stair and mobility sections; H/Ma: Hmax/Mmax ratio measured from the affected side; SD: Standard deviation; P25–P75: 25th–75th percentile; T1: Pretreatment; T2: Posttreatment; T3: Follow-up. Significance values have been adjusted by the Bonferroni correction for multiple tests. Bold values indicate statistical significance.

## References

[1] Wissel J Manack A Brainin M Toward an epidemiology of poststroke spasticity Neurology 2013 80 3 Suppl 2 S13 19 10.1212/WNL.0b013e3182762448 23319481

[2] Barnes MP An overview of the clinical management of spasticity Barnes MP Johnson GR Upper Motor Neurone Syndrome and Spasticity: Clinical Management and Neurophysiology 2nd ed Cambridge, UK Cambridge University Press 2008 1 8 10.1017/CBO9780511544866.002

[3] Struppler A Binkofski F Angerer B Bernhardt M Spiegel S A fronto-parietal network is mediating improvement of motor function related to repetitive peripheral magnetic stimulation: a PET-H_2_O^15^ study NeuroImage 2007 36 Suppl 2 T174 186 10.1016/j.neuroimage.2007.03.033 17499165

[4] Kinoshita S Ikeda K Yasuno S Takahashi S Yamada N Dose-response of rPMS for upper limb hemiparesis after stroke Medicine (Baltimore) 2020 99 24 e20752 10.1097/MD.0000000000020752 32541528 PMC7302622

[5] Struppler A Havel P Müller-Barna P Facilitation of skilled finger movements by repetitive peripheral magnetic stimulation (rPMS): a new approach in central paresis NeuroRehabilitation 2003 18 1 69 82 10.3233/NRE-2003-18108 12719622

[6] Krewer C Hartl S Müller F Koenig E Effects of repetitive peripheral magnetic stimulation on upper-limb spasticity and impairment in patients with spastic hemiparesis: a randomized, double-blind, sham-controlled study Archives of Physical Medicine and Rehabilitation 2014 95 6 1039 1047 10.1016/j.apmr.2014.02.003 24561057

[7] Chen S Li Y Shu X Wang C Ding L Electroencephalography mu rhythm changes and decreased spasticity after repetitive peripheral magnetic stimulation in patients following stroke Frontiers in Neurology 2020 11 546599 10.3389/fneur.2020.546599 33133002 PMC7550716

[8] Werner C Schrader M Wernicke S Bryl B Hesse S Repetitive peripheral magnetic stimulation (rpMS) in combination with muscle stretch decreased the wrist and finger flexor muscle spasticity in chronic patients after CNS lesion International Journal of Physical Medicine and Rehabilitation 2016 4 4 352 10.4172/2329-9096.1000352

[9] Ciortea VM Motoașcă I Borda IM Ungur RA Bondor CI Effects of high-intensity electromagnetic stimulation on reducing upper limb spasticity in post-stroke patients Applied Sciences 2022 12 4 2125 10.3390/app12042125

[10] Prouza O Kouloulas E Zarkovic D High-intensity electromagnetic stimulation can reduce spasticity in post-stroke patients International Journal of Physiotherapy 2018 5 3 87 91 10.15621/ijphy/2018/v5i3/173931

[11] Ruthiraphong P Sukhumvada T Wongphaet P Immediate effect of repetitive peripheral magnetic stimulation in hemiplegic patients with arm paresis: a pilot study ASEAN Journal of Rehabilitation Medicine 2021 31 1 16 22

[12] Fawaz SI Izumi SI Zaki AS Eldiasty SE Saadawy A Repetitive peripheral magnetic stimulation for improving upper limb function in post-stroke hemiparesis Egyptian Rheumatology and Rehabilitation 2023 50 1 35 10.1186/s43166-023-00204-x

[13] Beaulieu LD Massé-Alarie H Brouwer B Schneider C Noninvasive neurostimulation in chronic stroke: a double-blind randomized sham-controlled testing of clinical and corticomotor effects Topics in Stroke Rehabilitation 2015 22 1 8 17 10.1179/1074935714Z.0000000032 25776116

[14] Bohannon RW Smith MB Interrater reliability of a modified Ashworth scale of muscle spasticity Physical Therapy 1987 67 2 206 207 10.1093/ptj/67.2.206 3809245

[15] Johnson GR Pandyan AD The measurement of spasticity Barnes MP Johnson GR Upper Motor Neurone Syndrome and Spasticity: Clinical Management and Neurophysiology 2nd ed Cambridge, UK Cambridge University Press 2008 64 78 10.1017/CBO9780511544866.004

[16] Kinoshita S Ikeda K Hama M Suzuki S Abo M Repetitive peripheral magnetic stimulation combined with intensive physical therapy for gait disturbance after hemorrhagic stroke: an open-label case series International Journal of Rehabilitation Research 2020 43 3 235 239 10.1097/MRR.0000000000000416 32776765

[17] Grozoiu L Marin AG Ciobanu I Berteanu M Spastic tone increase reduced using repetitive peripheral magnetic stimulation: pilot study International Journal of Social Science and Humanity 2017 7 1 56 59

[18] Grozoiu L Simona S Hesse S Bighea A Berteanu M Repetitive peripheral magnetic stimulation in stroke rehabilitation: a case study International Journal of Social Science and Humanity 2016 6 8 608 611 10.7763/IJSSH.2016.V6.719

[19] El Nahas N Kenawy FF Abd Eldayem EH Roushdy TM Helmy SM Peripheral magnetic theta burst stimulation to muscles can effectively reduce spasticity: a randomized controlled trial Journal of Neuroengineering and Rehabilitation 2022 19 1 5 10.1186/s12984-022-00985-w 35034653 PMC8762845

[20] Krause P Edrich T Straube A Lumbar repetitive magnetic stimulation reduces spastic tone increase of the lower limbs Spinal Cord 2004 42 2 67 72 10.1038/sj.sc.3101564 14765138

[21] Nielsen JF Sinkjaer T Long-lasting depression of soleus motoneurons excitability following repetitive magnetic stimuli of the spinal cord in multiple sclerosis patients Multiple Sclerosis 1997 3 1 18 30 10.1177/135245859700300103 9160343

[22] Nielsen JF Sinkjaer T Jakobsen J Treatment of spasticity with repetitive magnetic stimulation: a double-blind placebo-controlled study Multiple Sclerosis 1996 2 5 227 232 10.1177/135245859600200503 9050361

[23] Serag H Abdelgawad D Emara T Moustafa R El-Nahas N Effects of para-spinal repetitive magnetic stimulation on multiple sclerosis related spasticity International Journal of Physical Medicine and Rehabilitation 2014 2 2 242 10.4172/2329-9096.1000242

[24] Grosse L Meuche AC Parzefall B Börner C Schnabel JF Functional repetitive neuromuscular magnetic stimulation (frNMS) targeting the tibialis anterior muscle in children with upper motor neuron syndrome: a feasibility study Children (Basel) 2023 10 10 1584 10.3390/children10101584 37892247 PMC10605892

[25] Grosse L Späh MA Börner C Schnabel JF Meuche AC Addressing gross motor function by functional repetitive neuromuscular magnetic stimulation targeting the gluteal muscles in children with bilateral spastic cerebral palsy: benefits of functional repetitive neuromuscular magnetic stimulation targeting the gluteal muscles Frontiers in Neurology 2023 14 1161532 10.3389/fneur.2023.1161532 37564737 PMC10410564

[26] Nito M Katagiri N Yoshida K Koseki T Kudo D Repetitive peripheral magnetic stimulation of wrist extensors enhances cortical excitability and motor performance in healthy individuals Frontiers in Neuroscience 2021 15 632716 10.3389/fnins.2021.632716 33679314 PMC7930341

[27] Zschorlich VR Qi F Schorer J Büsch D Sensory stimulation of the triceps surae muscle complex modulates spinal reflex responses: a comparison between tapotement massage and repetitive peripheral magnetic stimulation (rPMS) Brain Sciences 2024 14 2 119 10.3390/brainsci14020119 38391694 PMC10887412

[28] Behrens M Mau-Möller A Zschorlich V Bruhn S Repetitive peripheral magnetic stimulation (15 Hz rPMS) of the human soleus muscle did not affect spinal excitability Journal of Sports Science and Medicine 2011 10 1 39 44 24149293 PMC3737913

[29] Morozumi K Morishita K Tojima M Inomata T Effect of repetitive peripheral magnetic stimulation of the common fibular nerve on the soleus muscle Hoffmann reflex Journal of Physical Therapy Science 2024 36 5 278 283 10.1589/jpts.36.278 38694014 PMC11060763

[30] Matsuda T Kurayama T Tagami M Fujino Y Manji A Influence of peripheral magnetic stimulation of soleus muscle on H and M waves Journal of Physical Therapy Science 2018 30 5 716 718 10.1589/jpts.30.716 29765188 PMC5940480

[31] Burne JA Carleton VL O’Dwyer NJ The spasticity paradox: movement disorder or disorder of resting limbs? Journal of Neurology, Neurosurgery and Psychiatry 2005 76 1 47 54 10.1136/jnnp.2003.034785 15607994 PMC1739314

[32] Dietz V Sinkjaer T Spastic movement disorder: impaired reflex function and altered muscle mechanics The Lancet Neurology 2007 6 8 725 733 10.1016/S1474-4422(07)70193-X 17638613

[33] Hugos CL Cameron MH Assessment and measurement of spasticity in MS: state of the evidence Current Neurology and Neuroscience Reports 2019 19 10 79 10.1007/s11910-019-0991-2 31471769 PMC6948104

[34] Shemesh Y Rozin R Ohry A Electrodiagnostic investigation of motor neuron and spinal reflex arch (H-reflex) in spinal cord injury Spinal Cord 1977 15 3 238 244 10.1038/sc.1977.36 593714

[35] Katz RT Rovai GP Brait C Rymer WZ Objective quantification of spastic hypertonia: correlation with clinical findings Archives of Physical Medicine and Rehabilitation 1992 73 4 339 347 10.1016/0003-9993(92)90007-J 1554307

[36] Biering-Sørensen F Nielsen JB Klinge K Spasticity-assessment: a review Spinal Cord 2006 44 12 708 722 10.1038/sj.sc.3101928 16636687

